# Ecology of sound communication in fishes

**DOI:** 10.1111/faf.12368

**Published:** 2019-04-08

**Authors:** Friedrich Ladich

**Affiliations:** ^1^ Department of Behavioural Biology University of Vienna Vienna Austria

**Keywords:** calling activity, hearing, Lombard effect, noise, predator detection, sound characteristics

## Abstract

Fishes communicate acoustically under ecological constraints which may modify or hinder signal transmission and detection and may also be risky. This makes it important to know if and to what degree fishes can modify acoustic signalling when key ecological factors—predation pressure, noise and ambient temperature—vary. This paper reviews short‐time effects of the first two factors; the third has been reviewed recently (Ladich, 2018). Numerous studies have investigated the effects of predators on fish behaviour, but only a few report changes in calling activity when hearing predator calls as demonstrated when fish responded to played‐back dolphin sounds. Furthermore, swimming sounds of schooling fish may affect predators. Our knowledge on adaptations to natural changes in ambient noise, for example caused by wind or migration between quiet and noisier habitats, is limited. Hearing abilities decrease when ambient noise levels increase (termed masking), in particular in taxa possessing enhanced hearing abilities. High natural and anthropogenic noise regimes, for example vessel noise, alter calling activity in the field and laboratory. Increases in sound pressure levels (Lombard effect) and altered temporal call patterns were also observed, but no switches to higher sound frequencies. In summary, effects of predator calls and noise on sound communication are described in fishes, yet sparsely in contrast to songbirds or whales. Major gaps in our knowledge on potential negative effects of noise on acoustic communication call for more detailed investigation because fishes are keystone species in many aquatic habitats and constitute a major source of protein for humans.

## 1 INTRODUCTION

The ability to communicate effectively with other individuals plays a major role in the lives of all animals. Animals communicate under ecological constraints which may modify or hinder both signal transmission and detection or may be risky (Bradbury & Vehrencamp, 2011).

Representatives of several dozen families of bony fishes possess sound‐generating organs and vocalize (signal acoustically) in various behavioural contexts such as distress situations, agonistic interactions, courtship or to maintain group cohesion (Amorim, 2006; Fine & Parmentier, 2015; Ladich, 2015; Ladich & Bass, 2011; Ladich & Fine, 2006; Ladich & Myrberg, 2006; Myrberg, 1981; Myrberg & Lugli, 2006; Van Oosterom, Montgomery, Jeffs, & Radford, 2016). Sounds may also be emitted during other activities such as swimming in schools, and these swimming sounds may constitute acoustic signals too (Gray & Denton, 1991; Larsson, 2009; Moulton, 1960). It remains challenging to demonstrate the signal function experimentally. Moreover, an ever increasing number of auditory studies emphasize the importance of sound detection in bony fishes (Putland, Montgomery, & Radford, 2018; Sisneros, 2016). Despite this wealth of information on sonic organs, sounds and hearing abilities, our knowledge about the ecology of sound communication is very limited.

Among fishes, the acoustic detection of predators is the least investigated ecological constraint, whereas noise or more precisely noise pollution has become a hot topic (Cox, Brennan, Gerwing, Dudas, & Juanes, 2018; Popper & Hawkins, 2016; Putland, Merchant, Farcas, & Radford, 2017; Radford, Kerridge, & Simpson, 2014; Slabbekoorn et al., 2010). Anthropogenic noise is thought to endanger fish populations. To date, however, no effects on populations have been demonstrated in any study, in contrast to the damage done by overfishing (Jackson et al., 2001; Allan et al., 2005; www.greenpeace.org). A few contradictory observations are available regarding the effects of ship noise on calling behaviour (Correa et al., 2019; Luczkovich, Krahforst, & Sprague, 2012; Picciulin, Sebastianutto, Codarin, Calcagno, & Ferrero, 2012). The present review does not focus on anthropogenic noise as such, but on potential modifications of sound communication under varying noise conditions.

This paper reviews available empirical data regarding short‐time effects on sound communication in fishes when predators are detected acoustically and when background noise varies. Effects of ambient temperature have recently been reviewed (Ladich, 2018). Potential long‐time effects of ecological constraints on the evolution of sound communication in fishes are briefly discussed (Ladich, 2014a,b).

## 2 EFFECTS OF PREDATOR DETECTION ON SOUND COMMUNICATION

Sound traits that increase the attractiveness of males to females or help in assessing fighting abilities often make the senders more conspicuous to predators. In order to reduce the predation threat, communicating animals should be able to react to the presence of predators by modifying their signalling behaviour. Such modifications can include reducing the number of signals, modifying signal characteristics (e.g. by reducing sound levels), switching to different signalling channels or stopping signalling entirely. Killer whales (*Orcinus orca*, Delphinidae) pose a major threat to many whale species, making detecting their calls and responding accordingly crucial for survival (Cummings & Thompson, 1971; Fish & Vania, 1971).

Several studies have shown that fish respond to chemical and visual stimuli of predators and adjust their signalling during territorial, agonistic and courtship behaviour. Martel and Dill (1993) showed that juvenile coho salmon (*Oncorhynchus kisutch,* Salmonidae) significantly decreased their aggressive visual displays directed towards mirrors when exposed to chemical stimuli of an avian predator. Visually detected fish predator models (outside the tank) modified the fighting behaviour in the goldeneye cichlid (*Nannacara anomala,* Cichlidae). The cichlids preferred low‐intensity behaviours such as lateral display and tail beating because they are less dangerous and because they increase vigilance (Brick, 1998; Jakobsson, Brick, & Kullberg, 1995). Guppies (*Poecilia reticulata,* Poeciliidae) decreased conspicuous courtship displays and switched to visually less conspicuous sneak copulation when light intensity, and thus, the visibility of courting males was high (Endler, 1987; Magurran & Seghers, 1990).

Fish may be able to adapt acoustic signalling similar to changes in visual signalling when detecting predators. Acoustic signalling may be dangerous because predators can detect prey through passive listening. Gannon et al. (2005) demonstrated that bottlenose dolphins (*Tursiops truncatus*, Delphinidae) turn towards fish sounds. This helps explain why up to 80% of a dolphin's diet consists of soniferous fish species (Barros & Myrberg, 1987). This raises the question if and to what degree fishes adjust acoustic signalling when detecting predators so that they are less conspicuous but still able to communicate with conspecifics. A few studies revealed that fishes change their calling behaviour when hearing predator sounds. Male silver perch (*Bairdiella chrysoura,* Sciaenidae) diminished the loudness of mating choruses on average by 9 dB in their spawning areas when bottlenose dolphin signature whistles (3–4 kHz) were played back (Table 1) (Luczkovich et al., 2000). The authors suggested that individual fish ceased sound production near the sound source or simply swam away. The latter explanation implies that fish returned to their calling position within 1 min. In a subsequent study, Luczkovich and Keusenkothen (2007) played back dolphins signature whistles and echolocation sounds to longspine squirrelfish (*Holocentrus rufus*, Holocentridae). The results of that study were ambiguous; the authors concluded that fish appeared to vocalize less during the playback of dolphin sounds as compared to playbacks of conspecific calls and pure tones, although this was not supported statistically.

**Table 1:** Studies investigating the change in acoustic signalling when sounds of predators are played back. Behaviour of individual fish is unknown in all field studies

Species	Predator	Result	References
Silver perch (*Bairdiella chrysoura*, Sciaenidae)	Bottlenose dolphin, playback of signature whistles	Chorus loudness diminishes	Luczkovich et al. (2000)
Gulf toadfisch (*Opsanus beta,* Batrachoididae)	Bottlenose dolphin, playback of pop sounds	Decrease in calling activity	Remage‐Healey et al. (2006)
Longspine squirrelfish (*Holocentrus rufus,* Holocentridae)	Bott. dolphin, playback of signature whistles & echolocation sounds	Apparent decrease in calling activity	Luczkovich and Keusenkothen (2007)

**Figure 1:**
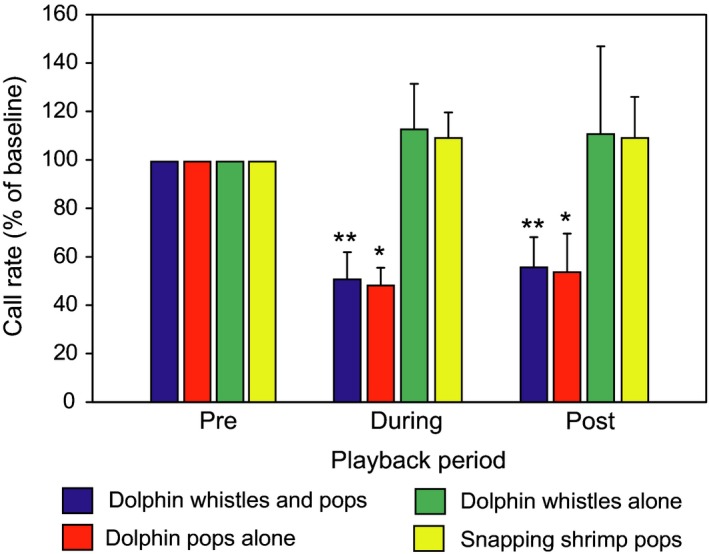
Mean (+*SEM*) changes in advertisement calling in male Gulf toadfish when different acoustic signals were played back. Redrawn after Remage‐Healey et al. (2006). Figure appears in colour in the online version only

Remage‐Healey, Nowacek, and Bass (2006) showed that playbacks of low‐frequency dolphin pop sounds (frequency range: 0.41–4.5 kHz), which are emitted by dolphins during foraging, reduced the nest advertisement calling rates in Gulf toadfish (*Opsanus beta,* Batrachoididae) by 50% in their natural environment (Figure 1). In parallel, the playbacks also elevated circulating stress hormone levels significantly. Gulf toadfish did not respond to high‐frequency (6–12.4 kHz) dolphin whistles which is in accordance with their inability to detect sound frequencies above 1 kHz (Fay, 1988; Ladich & Fay, 2013; Remage‐Healey et al., 2006; Sisneros, 2009). The lack of a response to snapping shrimp pops may be due to the playbacks of the uniform rate of pops emitted by shrimps in contrast to the highly variable rate of pops emitted by dolphins during foraging (Remage‐Healey et al., 2006; Thorson & Fine, 2002). Moreover, call duration did not change across treatment groups.

Both studies indicate that fishes do not modify sound characteristics when hearing a predator except that individual fish stop or reduce calling. These studies lack behavioural observations and leave the question open whether male fish modified signalling by reducing the level of their calls or whether the number of calling fish declined. It also remains unclear if fish are able to switch to other signal modalities.

Holt and Johnston (2009) attempted to answer these questions by choosing a small North American cyprinid as prey fish and sunfish and water snakes as predators. They tested if redeye bass (*Micropterus coosae*, Centrarchidae) and midland water snakes (*Nerodia sipedon* Colubridae) are attracted to the courtship sounds of tricolor shiner (*Cyprinella trichroistia*, Cyprinidae). Neither predator was attracted when sounds were presented alone. When visual signals were added, only the water snake responded more to the combined visual and acoustic signals than to visual signals alone. The lack of a response by red eye bass to sound playbacks could be due to the limited auditory sensitivity of the sunfish, which lack hearing enhancements (Wysocki & Ladich, 2005b).

According to Larsson (2009, 2012), schooling fish may actively manipulate predators acoustically by mimicking larger fish through their synchronized swimming sounds and hydrodynamic stimuli. This concept was termed the Acoustic Advantage Hypothesis. Based on this hypothesis, the most plausible and primary result of schooling (most advantageous effect for the prey) is that the individual fish will be hidden, undetectable by the predator's lateral line due to overlapping complex pressure waves from many adjoining fishes.

In summary, our knowledge about the effects of predator detection on acoustically communicating fishes is quite sparse despite the many studies showing behavioural changes in the presence of predators, for example in general activity (Tang, Huang, Wu, Kuang, & Fu, 2017), in foraging (reviewed by Milinski, 1993) as well as during social interactions (Brick, 1998; Endler, 1987). It is hypothesized that besides briefly reducing their calling activity, vocal species may switch to other signal modalities or to sneaking, or in schooling fish to actively misinforming predators. Increased visual signalling may not be an option because it may make the signaller more conspicuous to nearby predators. Territorial fish are in conflict between stopping advertising or fleeing their nest sites when they detect predators. The large number of vocal species found in the stomach of dolphins indicates that they may not hear the high‐frequency calls of dolphins or that predators hunt silently by intercepting fish sounds (Barros & Myrberg, 1987).

## 3 EFFECTS OF NOISE ON SOUND COMMUNICATION

Acoustic noise may affect sound communication in fishes in different ways (Ladich, 2013). First, noise of a particular level negatively affects hearing abilities in all fish species, a process termed masking. Longer lasting effects on hearing are known when fish are exposed to higher levels (e.g. Wysocki & Ladich, 2005a). The present review neglects such effects because it is unproven that they occur in the field in free‐swimming fish. Second, fish may try to overcome noise and adapt their calling behaviour, for example by modifying their sound characteristics accordingly. Third, fish may try to switch to other communication channels in the presence of noise, for example to visual or chemical channels.

### 3.1 Effect of noise on sound detection

Fay (1974) described increases in hearing thresholds (temporary threshold shift, TTS) in the goldfish (*Carassius auratus*, Cyprinidae) through masking when exposed to different levels of white noise. Such a threshold shift due to different noise types (white noise, natural ambient noise, anthropogenic noise) has subsequently been described in representatives of vocal and non‐vocal taxa of several non‐related fish families such as catfish, sunfish or croakers (Table 2) (Ramcharitar & Popper, 2004; Wysocki & Ladich, 2005b). Most studies measured hearing and masking in terms of sound pressure and only in a few species in terms of particle motion (Table 2). All fishes detect particle motion in a sound field at low frequencies (<1 kHz) and high sound levels. Numerous species evolved accessory hearing structures which enables them to transmit vibrations of gas‐filled cavities (e.g. swim bladder) to the inner ear. Accordingly, they can detect sound pressure and extend their hearing range up to several kHz and much lower sound levels (Ladich & Popper, 2004; Ladich & Schulz‐Mirbach, 2016; Popper, Fay, Platt, & Sand, 2003). It is technically challenging to prove that fish lacking a direct connection between gas‐filled cavities and the inner ear do or do not detect sound pressure (Popper & Fay, 2011).

**Table 2:** Studies investigating the temporal effects of noise (temporary threshold shifts, TTS) on hearing abilities due to masking

Species	Noise types	Result	References
Atlantic Cod (*Gadus morhua*, Gadidae)	Ambient noise in Scottish loch	TTS depending on sea noise level (SPL)	Chapman and Hawkins (1973), Chapman (1973)
Goldfish (*Carassius auratus*, Cyprinidae)	White noise playback, 3 noise levels	TTS varying with noise level (SPL)	Fay (1974)
Atlantic croaker (*Micropogon undulatus*, Sciaenidae)	White noise playback, 124 and 136 dB	TTS varying with noise level (SPL)	Ramcharitar and Popper (2004)
Black drum (*Pogonias chromis*, Sciaenidae)	White noise playback, 124 and 136 dB	TTS varying with noise level (SPL)	Ramcharitar and Popper (2004)
Common carp (*Cyprinus carpio*, Cyprinidae)	4 ambient noise types played back	TTS varying with noise level (SPL)	Amoser and Ladich (2005)
European perch (*Perca fluviatilis*, Percidae)	4 ambient noise types played back	TTS varying with noise level (SPL)	Amoser and Ladich (2005)
Goldfish (*Carassius auratus*, Cyprinidae)	White noise playback, 110 and 130 dB	TTS varying with noise level (SPL)	Wysocki and Ladich (2005b)
Raphael catfish (*Platydoras armatulus*, Doradidae)	White noise playback, 110 and 130 dB	TTS varying with noise level (SPL)	Wysocki and Ladich (2005b)
Pumpkinseed sunfish (*Lepomis gibbosus*, Centrarchidae)	White noise playback, 110 and 130 dB	TTS varying with noise level (SPL)	Wysocki and Ladich (2005b)
Lusitanian toadfish (*Halobatrachus didactylus*, Batrachoididae)	Ambient and ship noise playback	TTS in the presence of ship noise (SPL)	Vasconcelos et al. (2007)
Damselfish (*Chromis chromis*, Pomacentridae)	Ambient and ship noise playback	TTS in the presence of ship noise (SPL)	Codarin et al. (2009)
Brown meagre (*Sciaena umbra*, Sciaenidae)	Ambient and ship noise playback	TTS in the presence of ship noise (SPL)	Codarin et al. (2009)
Redmouth goby (*Gobius cruentatus*, Gobiidae)	Ambient and ship noise playback	TTS in the presence of ship noise (SPL)	Codarin et al. (2009)
Goldfish (*Carassius auratus,* Cyprinidae)	Pond and aquarium noise playback, 95–119 dB	TTS varying with noise level (SPL)	Gutscher, Wysocki, and Ladich (2011)
Orange chromide (*Etroplus maculatus*, Cichlidae)	White noise playback, 110 and 130 dB	TTS varying with noise level (SPL, PAL)	Ladich and Schulz‐Mirbach (2013)
Slender lionhead cichlid (*Steatocranus tinanti*, Cichlidae)	White noise playback, 110 and 130 dB	TTS varying with noise level (SPL, PAL)	Ladich and Schulz‐Mirbach (2013)

*Note*

Masking effects were determined in the presence of natural/ambient noise, ship/boat/aquarium noise and white noise. Experiments were carried out under controlled laboratory conditions except in the Atlantic cod. Sound levels were either determined as sound pressure levels (SPL) in dB re 1 μPa or as particle acceleration levels (PAL) in dB re 1 μms².

The masking effect strongly depends on the acoustic noise levels and absolute auditory thresholds. While the evolution of accessory hearing structures is unlikely linked to the evolution of sonic organs and sound communication (reviewed in Ladich, 2000, 2014a), it is safe to assume that noise affects fish depending on their hearing abilities. Masking effects are much more pronounced in species with excellent hearing abilities such as otophysines (carps and catfish) or some cichlids than in taxa lacking hearing enhancements (Ladich & Schulz‐Mirbach, 2013; Wysocki & Ladich, 2005a). The decline in hearing has seldom been measured in a fish's habitat. Chapman and Hawkins (1973) and Chapman (1973) measured hearing in the Atlantic cod (*Gadus morhua*, Gadidae) in a Scottish Loch. Hearing was only unmasked in calm sea conditions. Any change in sea noise level (different sea state) was accompanied by a shift in hearing thresholds. This threshold‐to‐noise ratio was constant at a particular frequency and increased from 18 dB at 50 Hz to 24 dB at 380 Hz. Fish will also encounter different noise levels, for example due to wind or when migrating between more quiet and noisier habitats. A study in the common carp (*Cyprinus carpio*, Cyprinidae) in which ambient noise from different habitats was played back revealed that its hearing is only moderately masked—as compared to quiet laboratory conditions—in standing waters (max. 9 dB TTS); it is heavily masked in flowing water such as rivers (up to 49 dB) (Amoser & Ladich, 2005).

Larsson (2009, 2012) pointed out that fishes’ hearing will be masked not only by ambient noise but also when swimming in schools due to the hydrodynamic noise created by their own movements and those of conspecifics nearby. Larsson (2009) suggested that synchronized movements and stopping locomotion simultaneously potentially reduces noise and the degree of masking for short periods.

**Figure 2:**
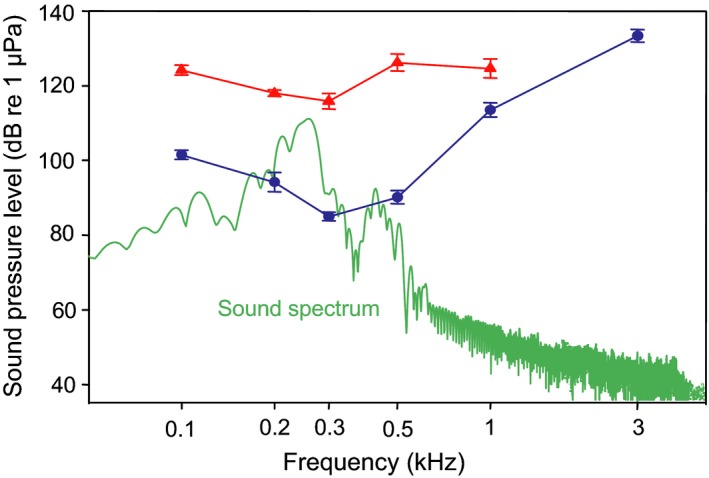
Mean hearing thresholds (±*SEM*) of the brown meagre measured during playback of natural ambient (circles) and boat noise conditions (triangles) compared to spectrum of conspecific knocking sounds. Modified from Codarin et al. (2009). Figure appears in colour in the online version only

All studies on sound transmission and calculations of active space therefore need to consider the effects of auditory masking of the particular species under particular noise conditions. Several studies illustrate this by comparing auditory thresholds measured during the playback of ambient noise and vessel noise to spectra of conspecific sounds (Scholz & Ladich, 2006; Vasconcelos, Amorim, & Ladich, 2007). Codarin, Wysocki, Ladich, and Picciulin (2009) showed in three Mediterranean species from different families that hearing thresholds did not change under either quiet laboratory or ambient noise conditions, but increased in the presence of boat noise by approximately 20 dB. Comparison of masked audiograms gained in the presence of boat noise to spectra of the brown meagre (*Sciaena umbra*, Sciaenidae) knock sounds revealed that the fish could no longer detect their sounds (Figure 2). Conservative calculations assuming a source level of 124 dB for sounds emitted by the brown meagre suggest that the distance at which conspecific sounds are detectable briefly decreases from 500 m under ambient noise conditions to about 1 m in the presence of boat noise (Codarin et al., 2009). Similar observations were made in the Lusitanian toadfish (*Halobatrachus didactylus*, Batrachoididae) indicating that acoustic communication is severely affected by masking of their hearing abilities (Vasconcelos et al., 2007).

In the New Zealand bigeye (*Pempheris adspersa*, Pempheridae), Van Oosterom et al. (2016) showed in laboratory experiments that shoals reduced the areas occupied in the presence of different levels (125, 130, 135 dB) of reef noise. This behaviour indicates that hearing is masked in New Zealand bigeye and that fish decrease interindividual distances to detect conspecific calls and communicate acoustically. It would be quite interesting to determine whether the degree of masking (in dB) is related to the shoal area occupied (in square metres). Another complementary mechanism may be that reef noise makes fish more agitated and stressed—a situation in which shoals tend to be more dense.

Road traffic noise can also mask hearing in freshwater fish that inhabit shallow streams close to roads. This makes detection of conspecific sounds even more challenging. Holt and Johnston (2015) investigated the impact of road traffic noise emanating from bridges on the transmission of vocalization in shallow streams. Playback of acoustic signals produced by the blacktail shiner (*Cyprinella venusta*, Cyprinidae) is masked at variable distances from the bridges depending on sound type, sound level, sound frequency and truck type. Their calculations reveal that growls and knocks will not be detectable close to bridges in the presence of truck noise. On average, knock sounds will emerge from truck noise with a signal‐to‐noise ratio (SNR) above 10 dB at a distance of 3 m at 172 Hz, whereas growls will emerge only 640 m from the bridge.

### 3.2 Effect of noise on calling activity

Some animals can adapt their calling activity to different noise regimes. This is known in amphibians but best studied in songbirds living in cities under varying levels of human‐made noise and in aquatic mammals, in particular whales (Brumm & Zollinger, 2013; Richardson, Greene, Malme, & Thomson, 1995; Sun & Narins, 2005). Interestingly, different ways to cope with noise apparently exist. Calling rates in anurans may increase or decrease under identical noise regimes such as airplanes (Sun & Narins, 2005). Whales may decrease their calling rates or lengthen their calls or songs in the presence of human‐made noise (Foote, Osborne, & Hoelzel, 2004; Lesage, Barette, Kingsley, & Sjare, 1999; Miller, Biassoni, Samuels, & Tyack, 2000; Richardson et al., 1995).

Aquatic noise varies considerably and typically increases in habitats or regions where water is moving fast or against barriers such as in rivers and streams, at waterfalls, at higher sea states or coastal surf (Kennedy, Holperied, Mair, Guzman, & Simpson, 2010; Urick, 1983; Wysocki, Amoser, & Ladich, 2007). I am unaware of any study showing that fish individuals adapt their calling behaviour in the field to different natural ambient noise levels. One such change, however, is known in the humpback whale (*Megaptera novaeanglia*, Balaenopteridae) (Dunlop, Cato, & Noad, 2010). The authors observed that humpback whales gradually switched from primarily vocal to primarily surface‐generated acoustic communication (breaching, pectoral fin slapping) when wind speeds and background noise levels increase.

**Figure 3:**
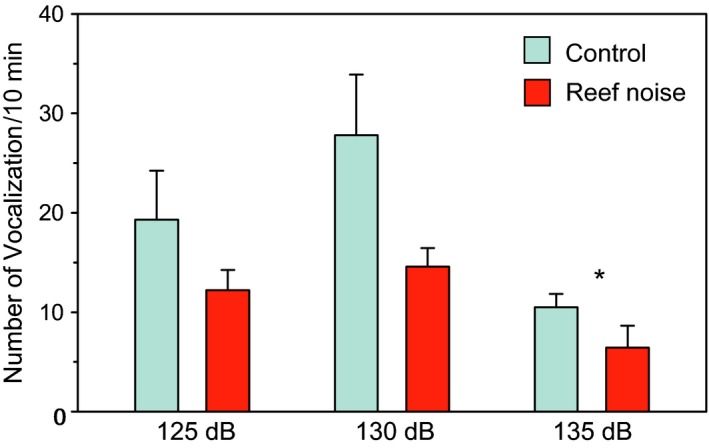
Mean (+*SEM*) number of vocalizations per 10 min of the New Zealand bigeye during silent control and when exposed to reef noise at three different levels (125, 130, 135 dB re 1 μPa). Modified from Van Oosterom et al. (2016). Figure appears in colour in the online version only

Van Oosterom et al. (2016) showed in laboratory experiments that the vocalization rate decreased in the New Zealand bigeye when reef noise was played back at the highest level (135 dB re 1 μPa) (Figure 3). De Jong, Amorim, Fonseca, Klein, and Heubel (2016) and De Jong, Amorim, Fonseca, Fox, and Heubel (2018) investigated the calling behaviour in the two‐spotted goby (*Gobiusculus flavescens*, Gobiidae) and the painted goby (*Pomatoschistus pictus*, Gobiidae) in laboratory experiments when a low‐frequency harmonic tone was added. They found that males of both species reacted to the noise (125 and 134 dB re 1 μPa, respectively). Courting males emitted fewer drum sounds than in the no‐noise treatment, whereas the number of thumps was not affected. This combined effect may have reduced the attractiveness of male painted gobies because females were less likely to spawn.

**Figure 4:**
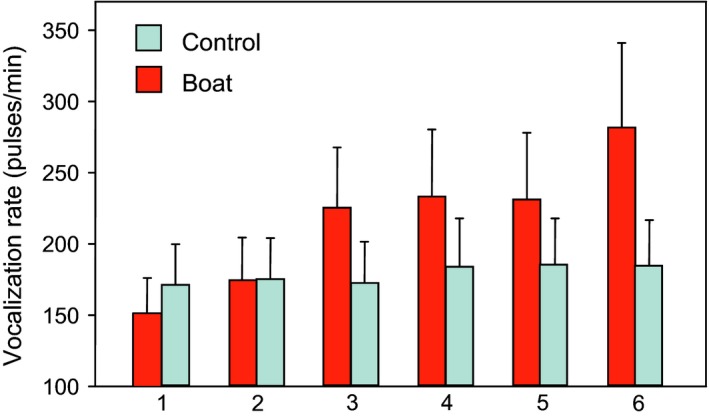
Mean (+*SEM*) vocalization rate of the brown meagre recorded after six boat passages (1–6). Note the increase in calling rate with the increase in the number of boat passages. Redrawn after Picciulin et al. (2012). Figure appears in colour in the online version only

A few field studies indicate that fish may modify their calling activity and sound characteristics in response to high anthropogenic noise levels. This information, however, requires support from additional studies that include visual observations. A short‐term increase in calling activity was recorded when different boat types passed over vocalizing brown meagre in a nearshore Mediterranean marine reserve (Table 3) (Picciulin et al., 2012). The fish's mean pulse rate increased after several boats passed in an experiment (Figure 4). Due to a lack of visual observations, we do not know whether the increase in calling rate is based on individual adaptations. Alternatively, it could be based on a higher number of fish being present and calling. Picciulin et al. (2012) argued that this behavioural change may be a case of vocal compensation for impaired intraspecific communication.

**Table 3:** Studies investigating the changes in acoustic signalling in the presence of natural and artificial (anthropogenic) noise types

Species	Noise	Result	References
Brown meagre (*Sciaena umbra*, Sciaenidae)	Boat noise passages	Mean pulse rate increased	Picciulin et al. (2012)
Atlantic croaker (*Micropogonias undulatus*, Sciaenidae)	Ferry noise in the field	No long‐term difference in calling rates	Luczkovich et al. (2012)
Blacktail shiner (*Cyprinella venusta*, Cyprinidae)	White noise playback in the laboratory	Produce shorter bursts and higher burst rates; spectral levels of growls and knocks higher	Holt and Johnston (2014)
Oyster toadfish (*Opsanus tau*, Batrachoididae)	Boat traffic noise in the field	Calling rates lower in high traffic areas	Luczkovich, Krahforst, Hoppe et al. (2016)
Two‐spotted goby (*Gobiusculus flavescens*, Gobiidae)	Low‐frequency harmonic tone playback in the laboratory at 134 dB	Males emit fewer courtship drums, thumps not affected	De Jong et al. (2016), De Jong, Amorim, Fonseca, Fox et al. (2018)
Painted goby (*Pomatoschistus pictus*, Gobiidae)	Low‐frequency harmonic tone playback in the laboratory at 125 dB	Males emit fewer courtship drums, thumps not affected	De Jong et al. (2016), De Jong, Amorim, Fonseca, Fox et al. (2018)
Oyster toadfish (*Opsanus tau*, Batrachoididae)	Vessel noise in the field	Call level increased by 7–9 dB	Luczkovich, Krahforst, Kelly et al. (2016)
New Zealand bigeye (*Pempheris adspersa*, Pempheridae)	Reef noise playback in the laboratory at 125, 130, 135 dB	Calling rates decrease at 135 dB noise level	Van Oosterom et al. (2016)

Luczkovich et al. (2012) investigated sounds emitted by the Atlantic croaker (*Micropogonias undulatus*, Sciaenidae) using a long‐term acoustic recording system in the Pamlico River estuary, North Carolina, from March to December. Sound production was typically associated with spawning but not with ferry passages over the course of several months. The passage of large vessels appeared to briefly reduce calling activity. A second long‐term data recording was carried out by these investigators in the Neuse and Pamlico River to determine whether oyster toadfish (*Opsanus tau*, Batrachoididae) are disturbed by vessels (Luczkovich, Krahforst, Hoppe, & Sprague, 2016). Calling rates were lower in the high‐boat traffic area (Neuse River) than in the low vessel‐noise site (Pamlico River). The authors assumed that toadfish could not call over the loud vessel noise and therefore reduced their calling rate. In order to confirm that oyster toadfish reduced calling rates at particular noise levels, the replication of experiments across different sites will be necessary. In summary, field observations are contradictory inasmuch as they have reported increases, decreases or even no change in calling activity in the presence of noise. The results may depend on the different noise conditions, behavioural contexts or reflect species‐specific differences in response to noise.

### 3.3 Effects of noise on sound characteristics

Optimizing sound communication in noise can involve changes in calling rate and call duration but may also include a shift in frequency bands and increase in sound level (Lombard effect).

#### 3.3.1 Effects of noise on call frequencies

Anthropogenic noise typically consists of low frequencies, and songbirds and whales may shift to higher frequencies to facilitate acoustic communication (Brumm & Zollinger, 2013; Lesage et al., 1999; Tyack, 2008). No reports are available showing that fish can shift their frequency band of communication in noise.

Nevertheless, a correlation between sound frequencies and ambient noise spectra to optimize acoustic signalling has been proposed in noisy aquatic environments. Most vocal fish produce low‐frequency sounds with main energies between approximately 100 and 300 Hz. This typically involves rapid contractions of sonic muscles (Ladich & Fine, 2006; Lugli, 2015). Such low sound frequencies often correlate to quiet windows in ambient noise in the fish's habitats (Holt & Johnston, 2015). Italian freshwater gobies such as the Padanian goby (*Padogobius martensii*, Gobiidae) and Arno goby (*Gobius nigricans*, Gobiidae) emit sounds with main frequencies in the 80–200 Hz band which fall within a quiet low‐frequency band in streams in northern Italy (Lugli & Fine, 2003, 2007; Lugli, Yan, & Fine, 2003). Lugli (2010) extended his conclusions to other gobies, toadfishes (family Batrachoididae), sculpins (family Cottidae), minnows (family Cyprinidae) and darters (family Percidae) when comparing mating sounds to frequency bands of quiet windows. A correlation at higher frequencies between 300 Hz and 2 kHz was described in the weakly electric mormyrid *Pollimyrus isidori* (Mormyridae) inhabiting the Niger River in Mali (Crawford, Jacob, & Benech, 1997). In contrast, Coers, Bouton, Vincourt, and Slabbekoorn (2008) reported that the ambient noise in a tidal zone was most pronounced at the communication frequencies of rock‐pool blennies (*Parablennius parvicoris*, Blenniidae). In summary, while a correspondence between sound frequencies and noise windows is found in several fishes, no proof of fitness advantages, and therefore, no evidence for an adaptation is available yet.

#### 3.3.2 Lombard effect

Birds very flexibly adjust their songs as ambient noise levels vary. Free‐ranging nightingales (*Luscinia megarhynchos*, Muscicapidae), for example, sing louder during working days than on weekends when there is less traffic (Brumm, 2004). The Lombard effect is a well‐known basic vocal mechanism in birds and mammals for acoustic communication in noise (Brumm & Zollinger, 2011; Holt, Noren, Veirs, Emmons, & Veirs, 2008; Scheifele, Andrew, Cooper, & Darre, 2005).

**Figure 5:**
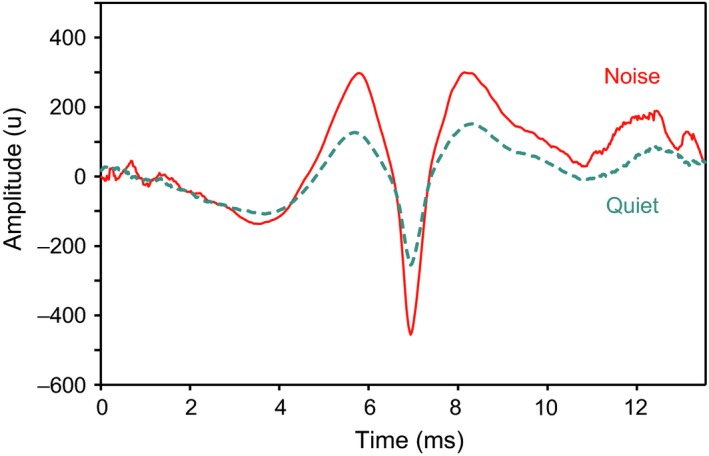
Oscillograms illustrating higher amplitude of communication sound pulses of the blacktail shiner in the presence of white noise. Pulses were constructed by averaging 373 pulses produced under noisy conditions (solid line) and 403 pulses produced under quiet conditions (broken line). All sounds were produced by the same individual. Redrawn after Holt and Johnston (2014). Figure appears in colour in the online version only

Holt and Johnston (2014) studied the effect of elevated noise levels on acoustic signals and inter‐fish distance in blacktail shiners*,* which inhabit streams in the south‐eastern United States. Shiners altered several characteristics of their growling and knocking sounds under noisy condition in the laboratory. The number of pulses per growling call decreased (yielding shorter bursts) and the burst rate increased, demonstrating for the first time that individual fish may change temporal patterns of their calls. The most interesting outcome of that study was that spectral levels of both growls and knocks were significantly higher under noisy conditions. This also indicated for the first time the presence of a Lombard effect in fishes (Table 3) (Figure 5). Spectral levels increased by 5 to 10 dB depending on the frequency and sound type. In any case, these values were lower than the increase in background noise levels at these frequencies (10 dB to 16 dB). The authors hypothesized that blacktail shiners try to compensate for the decreased signal‐to‐noise ratio by increasing the sound level (Lombard effect) and number of signals. In contrast to their expectations, the blacktail shiner produced fewer pulses per call under noisy conditions. This most likely decreased the overall amount of information transmitted to conspecifics during aggressive or reproductive activities.

The Lombard effect was also indicated in a field study on the oyster toadfish's advertisement calls. Luczkovich, Krahforst, Kelly, and Sprague (2016) played back vessel noise and recorded SPLs (expressed as call power in dB re 1 μPa²) of vocalizations emitted by oyster toadfish. The average call level increased by 7 and 9 dB during and after playback periods relative to pre‐period level. No switch in the fundamental frequency was observed. Oyster toadfish males clearly attempted to compensate masking by noise. These two studies on unrelated taxa (freshwater cyprinid vs. marine batrachoidid) utilized quite different experimental set‐ups (white noise in laboratory and measurements of individual fish vs. ship noise in the field and lack of individual data). They support the notion that a noise‐dependent regulation of vocal amplitude (Lombard effect) exists at least in some vocal fish, a behaviour which was up until recently attributed solely to amniotes (birds and mammals) (Brumm & Zollinger, 2011).

### 3.4 Noise and compensation of acoustic communication by other signalling modalities

Fish may compensate potential limitations in acoustic signalling due to masking by noise by switching to other signal modalities, in particular visual signalling. In this way, they may be able to deter opponents and attract mates similarly as in low noise conditions so that their reproductive success is not hampered. The Bornea rock frog (*Staurois parvus*, Ranidae), for example, lives permanently under the continuous noise of running water. They use conspicuous visual signals such as foot‐flagging in addition to their calls as advertisement signals (Grafe et al., 2012). Nonetheless, it remains to be investigated whether the Bornea rock frog refrains from visual displaying in quiet environments.

So far, there is no evidence that vocalizing fish signal visually more in the presence of acoustic noise. De Jong, Amorim, Fonseca, Fox et al. (2018) observed that male painted gobies vocalized less under noisy conditions. This went hand in hand with fewer visual courtship displays, which reduced the probability of spawning. This is opposite to the expected behaviour and undermines the hypothesis that signalling fish compensate the loss in acoustic information by other signal modalities. While there is no evidence of compensation on the part of the male sender, female receivers paid more attention to visual courtship signalling in the noise experiments (sensory compensation hypothesis; Partan, 2017; De Jong, Amorim, Fonseca, & Heubel, 2018). This is because the females probably did not perceive male courtship calls at all due to masking of their already low hearing abilities (Lugli & Fine, 2003).

While experimental evidence for a compensation of acoustic signals by other signal types (visual. hydrodynamic/lateral line, chemical) is lacking, whether sound communication evolved under particular ecological conditions has frequently been discussed. Darkness or lack of visibility may have resulted in the evolution of sound generation. While this evolutionary scenario cannot be excluded, it is not supported by the majority of behavioural observations. These show that fish typically start vocalizing after conspecifics have been detected visually in agonistic and reproductive contexts and that fish rarely respond to chemical or acoustic signals alone (Chabrolles et al., 2017; Ladich, 1989, 1998; Lugli & Fine, 2003). Thus, acoustic signals are not produced primarily to compensate visual signals but typically in support of visual signals when intruders need to be threatened or mates attracted into nests.

### 3.5 Noise and effects on communication range

Distances over which fish communicate acoustically (active space) will potentially decrease when ambient noise levels increase at frequencies used for communication. So far, no playback experiments have proved that fish detect and respond to sound sources at shorter distances in the presence of noise. Several studies estimated the communication space based on particular models, on natural ambient and anthropogenic noise levels, and on hearing sensitivities and sound levels. They calculated a considerable decrease in distance in the presence of various anthropogenic noise sources (e.g. Putland et al., 2017; Stanley, Parijs, & Hatch, 2017; Van Oosterom et al., 2016; Vasconcelos et al., 2007). These calculations, however, need to be treated cautiously because none of them provide baseline communication distances or additional evidence that fish maintain constant sound levels. Such calculations always require confirmation by phonotaxis experiments either in the laboratory or in the field, showing that fish approach speakers differently depending on noise.

Sound attenuation can reach 30–40 dB at 10 m from the sound source, for example in natural shallow waters (Alves, Amorim, & Fonseca, 2016; Fine & Lenhardt, 1983). This is mainly due to the cut‐off frequency phenomenon which limits the propagation of long wavelengths in shallow water (Forrest, Miller, & Zagar, 1993; Lugli, 2015; Rogers & Cox, 1988). Fish largely communicate by using low‐frequency (<1 kHz) sound—a major limitation of fish communication compared with terrestrial animal and whale communication (Bass & Clark, 2003; Ladich & Winkler, 2017). The proven maximum communication distances in fishes are approximately 10 m and on average much smaller (see above) (Amorim, Vasconcelos, Bolgan, Pedroso, & Fonseca, 2018; Lugli, 2015; Mann, 2006). Lugli and Fine (2003) noted that call levels of goby sounds (90–120 dB at 5–10 cm) are below the noise level 50–60 cm from the source, even under quiet conditions. Typically, males will start calling after females are detected visually and enter the nest site, a process occurring at a distance of a few centimetres.

In addition, fish can increase the amplitude of their calls (Lombard effect) and may partly be able to maintain their communication ranges, as has been demonstrated in numerous studies in songbirds and whales and recently in fish (Holt & Johnston, 2014; Parks, Johnson, Nowacek, & Tyack, 2011).

## 4 SUMMARY AND FUTURE RESEARCH

Our current knowledge of the influence of various ecological constraints on sound communication in fish is large in the case of temperature but very limited and patchy in the case of noise and hearing of predators. The question whether noise affects predator detection is entirely unexplored.

We know nearly nothing about the effects of predation threats on calling behaviour in fishes. The available data suggest that prey fish stop calling after detecting dolphin sounds, but nothing is known about their responses in the presence of piscivorous fish. The acoustic advantage hypothesis postulates that schooling fish produce sounds via synchronous swimming which mimics large fish and thus misinforms predators. Future experiments should observe vocalizing fish individually to determine whether they can modify sound properties besides calling rates. One alternative is to switch to other signalling modalities or, similar to courting guppies, from elaborate signalling to sneaking behaviour.

The effects of noise on calling activity and signal characteristics are difficult to interpret because experimental set‐ups are not comparable, visual observations in the field are lacking and the results are contradictory. Although fish live in a large variety of natural ambient noise conditions, nearly nothing is known on the effects of these conditions on sound communication. Only a few auditory studies have investigated natural noise conditions, and our knowledge is mostly based upon the effects of anthropogenic noise. Playbacks of reef noise in the laboratory indicate that high levels reduced calling activity in bigeyes as well as in gobies in the presence of low‐frequency, continuous harmonic noise (De Jong et al., 2016; De Jong, Amorim, Fonseca, Fox et al., 2018; Van Oosterom et al., 2016). When boats passed by, for example, the calling rate in drums increased and sound production in oyster toadfish briefly decreased in the field (Luczkovich, Krahforst, Hoppe et al., 2016; Picciulin et al., 2012). These different outcomes may reflect the different noise types, noise levels and noise durations applied and the fish species investigated. Unambiguously, describing the responses of fish necessitates individual observations in the presence of different noise types using video recordings under standardized conditions.

There are some indications that fishes can modify signal characteristics. Changes in sound characteristics have been observed in the presence of noise, but many more data are necessary to confirm this. Fishes can apparently call louder in noisy conditions and modify temporal patterns. Changes in call frequencies, however, seem to be absent in fishes, in contrast to toothed and baleen whales where an increase call frequencies were reported when vessel noise increases. Future experiments should focus on individual fish and less on aggregations. Such experiments will help to clarify the question whether the changes in sound properties observed so far are robust and comparable to other animal taxa. For example, fishes and frogs seem to be unable to switch to different signal modalities under noisy conditions. This conclusion, however, may be premature due to the small amount of experimental data given.

In summary, there are major gaps in our knowledge on the effects of acoustic factors (predator calls, noise and the interaction between both) on sound communication in fishes. Currently, it is only tentatively possible to compare fishes to songbirds and whales. Potential negative effects of sound on acoustic communication need to be investigated because fishes are keystone species in many aquatic habitats and constitute a major source of protein for humans.
